# Supplementary material for: Prevalence and incidence of Coats disease in a large claims database
Source: PLoS One. 2025 Dec 29;20(12):e0329871. doi: 10.1371/journal.pone.0329871 (PMC12747393; doi:10.1371/journal.pone.0329871)
Supplement: Attachment — Submitted filename: Response to Reviewers.docx [file pone.0329871.s002.docx]

**Thank you for taking the time to review our manuscript and for providing thoughtful comments. We have revised our manuscript by incorporating all comments.**

**Editor:**

**The grant information has been removed from the Acknowledgements Section. The following sentence has been added the on-line statement part: “JSPS KAKENHI (grant number 24K12756) for NO”.**

**Regarding the Public Sharing of Raw Data (the dataset used for analysis in this study):**

**The data used in this study were obtained from the JMDC Claims Database under a contractual agreement between JMDC Inc. and the University of Occupational and Environmental Health, Japan. Because the terms of this agreement prohibit the authors from sharing the data with third parties, the dataset cannot be made publicly available.**

**However, as the dataset is commercially available for purchase from JMDC Inc., the authors did not have any special access privileges that others would not have. Other researchers may access the same dataset directly from JMDC Inc. by paying the required usage fee. For inquiries regarding data access, please contact JMDC Inc. through their website:**

**(Japanese) https://www.jmdc.co.jp/inquiry/**

**(English) https://www.jmdc.co.jp/en/inquiry/**

**Reviewer #1:**

*In the ≥20-year-old group in particular, the study population is biased because the JMDC database primarily includes employees enrolled in corporate health insurance and their dependents. This results in underrepresentation of elderly individuals (especially those ≥75 years), self-employed persons, and the unemployed. As the age, occupational, and socioeconomic distributions differ from those of the general population, adjustments such as age standardization are recommended to produce more accurate, population-based estimates.*

**[Answer] Thank you for the valuable comment. We have changed the prevalence and incident data to age-adjusted data in Abstract, text and Tables 3-6. Accordingly, the detail of the adjustments was added in the Methods section: “Sex-, age group– (children and adults), and overall prevalence and incidence rates were age-adjusted using the 2015 standard population of Japan provided by the Ministry of Health, Labour and Welfare”. Additionally, we have added the following sentence to lines 300–301 of the paragraph discussing bias in the study population: “The age distribution of the study population was adjusted to the age structure of the standard Japanese population.”**

**Reviewer #2:**

*Minor points;
1. Absence of Age-Adjusted Prevalence:
While the study presents age-stratified prevalence rates, it does not provide an age-adjusted prevalence rate standardized to a reference population (e.g., the general Japanese population). The age structure of the JMDC database (75% adults, 25% children) likely differs from that of the general population in Japan. Therefore, the calculated crude prevalence rate (2.1/100,000) may be influenced by the specific age demographics of the database. Presenting an age-adjusted rate would enhance the generalizability of the findings and allow for more accurate comparisons with other populations.*

**[Answer] Thank you for the valuable comment. We have changed the prevalence and incident data to age-adjusted data in Abstract, text and Tables 3-6. Accordingly, the detail of the adjustments was added in the Methods section: “Sex-, age group– (children and adults), and overall prevalence and incidence rates were age-adjusted using the 2015 standard population of Japan provided by the Ministry of Health, Labour and Welfare”. Additionally, we have added the following sentence to lines 300–301 of the paragraph discussing bias in the study population: “The age distribution of the study population was adjusted to the age structure of the standard Japanese population.”**

*2. Limitations of Case Definition:
The study identifies patients based solely on the standardized diagnostic name "Coats disease". The authors should more explicitly discuss the limitations of this approach in the Discussion section. There is a potential for misclassification. Acknowledging limited diagnostic accuracy, without validation analysis, is a key limitation of the study.*

**[Answer] Thank you for the valuable suggestion. The following statement has been added in the limitation section:**

**“Third, in this study, patients were identified solely based on the standardized disease name “Coats’ disease.” Because Coats’ disease is typically diagnosed in childhood and presents with characteristic fundus findings, the diagnostic coding accuracy is considered to be high. However, as the database contains only coded information, it is difficult to verify potential misclassification for each case as we did in our own clinical cases. Therefore, the possibility of case misclassification cannot be completely excluded in this study.”**

*3. Definition of Treatment Rate:
The definition of treatment is restricted to retinal photocoagulation and vitrectomy. While these are primary interventions, adjunctive therapies such as intravitreal anti-VEGF injections are now common. It should be clarified in the limitations that the reported "treatment rate" specifically reflects the rate of major surgical interventions and may not capture all therapeutic modalities.*

**[Answer] Thank you for the valuable suggestion. The following statement has been added in the limitation section as well: “Forth, the treatment options selected here were retinal photocoagulation and vitrectomy. However, adjunctive therapies such as intravitreal injection of anti-VEGF agents are also currently used as off-label treatments [36]. Therefore, the treatment rate in this study reflects the proportion of standard interventions and does not include all treatment modalities.”**
